# A consensus-weighted multi-agent ensemble feature selection framework for antibiotic susceptibility prediction in pediatric respiratory diseases

**DOI:** 10.3389/frai.2026.1922227

**Published:** 2026-09-22

**Authors:** Sarlinraj Madhalaimuthu, Sujatha Radhakrishnan

**Affiliations:** School of Computer Science Engineering and Information Systems, Vellore Institute of Technology, Vellore, Tamil Nadu, India

**Keywords:** consensus feature selection, interpretable models, pediatric antibiotic susceptibility, SHAP analysis, soft voting ensemble

## Abstract

**Background:**

Antibiotic susceptibility prediction is important because of the risks of failing to respond to antibiotics and the risk of antimicrobial resistance when treating respiratory diseases in children. Machine learning models can represent complex relationships among clinical and microbiological data; however, their effectiveness is strongly dependent on interpretability, stability, and methodological robustness. Redundancy-aware consensus-based feature selection frameworks provide a reliable way to enhance predictive performance and clinical usability.

**Methods:**

Preprocessing, balancing, leakage-controlled splitting of integrated pediatric clinical and nasopharyngeal antibiogram data, and binary antibiotic susceptibility prediction were conducted. Consensus scoring, vote strength, and redundancy penalization were used to create a model by combining six feature selection agents: Random Forest importance, Gradient Boosting importance, ANOVA F-test, Mutual Information, RFE-Random Forest and RFE-Gradient Boosting. A total of twelve classifiers were tested, and the three best classifiers were selected using a genetic algorithm for soft-voting ensemble construction. Standard metrics, cross-validation, statistical tests, and SHAP explainability were employed to validate model performance.

**Results:**

In predicting antibiotic susceptibility, LightGBM achieved the highest accuracy of 0.8718, the highest F1-score of 0.8726, and the highest ROC-AUC of 0.9498. Optimal models for soft voting ensemble construction were identified using a genetic algorithm: Decision Tree, XGBoost, and LightGBM. The ensemble model attained an accuracy of 0.8799, recall of 0.8907, specificity of 0.8692, F1-score of 0.8811, and ROC-AUC of 0.9490.

**Discussion:**

The proposed consensus-based multi-agent feature selection method successfully selected a compact, non-redundant, and clinically interpretable predictor subset for predicting antibiotic susceptibility in pediatric patients. Antibiotic identity and bacterial organism were primary factors in SHAP analysis, with clinical, inflammatory, environmental, and hospital-related factors also playing significant roles. The framework offers a meaningful and statistically sound machine learning tool for studies in pediatric antimicrobial decision support.

## 1 Introduction

Artificial intelligence (AI) and machine learning (ML) are being used in clinical prediction modeling to analyze data in complex healthcare situations and are increasingly applied for clinical diagnostics, prognosis, and decision support. However, achieving predictive accuracy alone is insufficient for translating models into clinical applications. The study emphasizes that robust prediction models should be translatable, thoroughly evaluated, reproducible, and have clear definitions of sources, outcomes, model development, validation, and clinical applicability (Collins et al., 2024). Furthermore, AI clinical decision-support systems must be assessed early in terms of clinical performance, safety, usability, and human factors before deployment in healthcare environments (Vasey et al., 2022). Thus, transparency and methodological rigor are crucial for building AI systems that are scientifically valid and can support clinical decision-making without replacing it.

Optimal antibiotic selection is a significant clinical challenge; respiratory tract infections are a major factor associated with antibiotic usage in pediatric populations. Pediatric acute respiratory tract infection (ARTI) trials suggest that treatment with broad-spectrum antibiotics is not associated with better clinical or patient-centered outcomes than narrow-spectrum antibiotics and is linked to more adverse events (Gerber et al., 2017). Biomarkers like C-reactive protein and procalcitonin can provide supportive information in pediatric pneumonia to help differentiate between bacterial and viral pneumonia, determine disease severity, and support antibiotic stewardship, but they should not be used independently of clinical findings (Omaggio et al., 2024). These challenges underscore the need for data-driven strategies that incorporate clinical, microbiological, laboratory, and antibiotic-related variables to aid in making more accurate antimicrobial decisions.

Machine learning offers a pragmatic approach to modeling the complex, non-linear relationships between diverse clinical and microbiological variables in the context of antimicrobial resistance (AMR) and antibiotic susceptibility prediction. For instance, models based on XGBoost have shown moderate predictive performance for Enterobacterales bloodstream infections at the time of the first blood culture sample, with better performance observed after the organism had been identified, highlighting the value of pathogen-specific information (Yuan et al., 2025). Likewise, multimodal EHR-based methods have demonstrated the benefits of combining structured clinical and laboratory data with textual information to improve AMR prediction (Hardan et al., 2024), and studies at the hospital level have shown the potential of ML methods to use historical resistance patterns, antimicrobial use, and interactions between pathogens and antimicrobials for predictive tasks (Vihta et al., 2024). Despite these advances, reliable, stable, and clinically interpretable predictor selection is not guaranteed by the ability to predict.

Biomedical prediction datasets generally have high dimensionality and heterogeneity, consisting of variables based on clinical observations, laboratory measurements, microbiological data, treatment data, and coded categorical descriptors. These datasets typically contain multiple redundant and correlated predictors that may lead to increased dimensionality, overfitting, and poor clinical meaningfulness of the model. Recent research on feature selection in healthcare highlights the significant challenges of handling high-dimensional datasets for clinical analysis, emphasizing the need for feature selection to reduce dimensionality, enhance computational efficiency, and improve interpretability (Maruotto et al., 2025). Furthermore, in addition to predictive accuracy, feature selection should also consider feature stability, cross-method similarity, and clinical relevance (Wang et al., 2025). Combining complementary methods can enhance the robustness of ensemble feature selection techniques, but some simple aggregation approaches, like majority voting, may fail to capture the complementarity between features and manage redundancy (Battistella et al., 2024). Consensus-based and redundancy-aware feature selection is motivated by these limitations for antimicrobial susceptibility prediction.

Beyond its strong capabilities in feature selection, explainable artificial intelligence (XAI) is a key element in ensuring that machine learning models are clinically meaningful and interpretable. SHapley Additive exPlanations (SHAP) is an increasingly popular *post hoc* interpretability technique, especially when combined with tree-based models, due to its ability to provide local and global explanations of model predictions (Caterson et al., 2024). In terms of methodology, SHAP provides a common, additive mechanism for feature attribution, assigning the contribution value of a prediction to each predictor in a theoretically grounded way (Lundberg and Lee, 2017). SHAP facilitates the interpretation of individual predictions and the evaluation of the overall importance of features and feature–outcome relationships, which is useful for healthcare applications. However, it is crucial to keep in mind that SHAP values are not causal effects but rather model attribution effects and should thus be interpreted with care.

### 1.1 Research objectives and contributions

This study is aimed at developing an interpretable consensus-weighted multi-agent feature selection framework for predicting antibiotic susceptibility in pediatric patients based on integrated clinical and nasopharyngeal aspirate antibiogram data. The goal is to identify a small, interpretable, and non-redundant subset of predictors useful for the binary classification of susceptibility, with an emphasis on clinical relevance, robustness, and applicability to explainable machine learning methods. Moreover, the study assesses the predictive power and applicability of the selected features to various classification models.

This study aims to:

1. Combine patient clinical data, bacterial data, and standardized antibiotic data into a more complete and diverse dataset for predicting pediatric susceptibility to antimicrobials, incorporating both host and pathogen-related variability in antimicrobial response.
2. Test the relevance of the predictors from several methodological perspectives using six distinct feature selection agents—both embedded and filter-based, as well as wrapper-based—to avoid the bias of single-method feature selection.
3. Calculate a ranking of agents based on a consensus-driven approach that includes consensus scoring, vote strength, and redundancy penalization, with the intention of identifying agents that are stable (exhibiting low variance in their ranks across iterations), non-redundant (differing from others in the ranking), and have high utility (achieving high scores on the consensus ranking).
4. Test and compare the predictive capacity of the feature subset selected by multiple baseline machine learning classifiers, while improving the predictive capacity of the classifiers by optimizing the combination of classifiers using a genetic algorithm-based weighted soft-voting ensemble.
5. Evaluate the predictive models using statistical validation methods and explainability via SHAP to understand the features’ contributions to the model and to provide both global and local interpretations.

The foremost contribution of this work is the introduction of a consensus-based multi-agent feature selection algorithm with redundancy awareness for pediatric antibiotic susceptibility prediction. This study differs from methods that solely aim for accurate predictions, as it integrates multi-agent feature selection with redundancy control, ensemble learning, statistical validation, and EAI into a unified and interpretable biomedical machine learning pipeline.

### 1.2 Scientific importance of the study

The significance of this study is that it advances from accuracy-centric solutions to a more transparent, robust, and interpretable biomedical machine learning pipeline for antibiotic susceptibility prediction. However, in the healthcare prediction context, high performance is insufficient unless the selected predictors are reliable, non-redundant, and carry clinical significance. To address this gap, the present work introduces a novel framework that combines consensus-based feature selection, redundancy-aware ranking, ensemble learning, statistical validation, and explainability of the learning model using SHAP. This study proposes a unified pediatric antibiotic susceptibility prediction pipeline that incorporates consensus-based feature selection, redundancy-aware ranking, ensemble learning, statistical validation, and explainability of the learning model using SHAP. Through model selection and interpretable feature analysis, the workflow provides a structured methodological framework for pediatric antimicrobial susceptibility prediction research. Its potential applicability to clinical decision support requires confirmation through independent external and prospective validation.

## 2 Literature review

### 2.1 Machine learning for antimicrobial resistance and antibiotic susceptibility prediction

The complex relationship between pathogens, host factors, and treatment history creates the need for machine learning in the field of antimicrobial resistance (AMR) and antibiotic susceptibility prediction. Non-linear relationships among clinical and microbiological data are captured in models to aid in early therapeutic decisions. Performance is moderate when considering the timing of culture collection, but with organism-level information, it improves, making it a more effective predictor. However, species identification is not always available in a timely manner, complicating immediate empirical decision-making (Yuan et al., 2025). Previous studies have used machine learning to analyze hospital electronic medical records for predicting antibiotic resistance in an ensemble model. Integrated modeling strategies, such as logistic regression, gradient-boosted trees, and neural networks, have demonstrated value, with improved predictive performance observed through their combination. Model accuracy was further enhanced with the introduction of bacterial species identity, underscoring the importance of not only prior resistance history but also this variable in modeling antibiotic resistance (Lewin-Epstein et al., 2021).

Large-scale antimicrobial susceptibility and surveillance datasets are used to evaluate machine learning models for AMR prediction. Gradient boosting classifiers performed well among the classifiers, with particularly strong performance in phenotype-only and combined datasets. Antibiotic identity is highlighted as a key factor in the feature importance and explanation methods, underlining the significance of antibiotic-specific information in the susceptibility modeling process (Valavarasu et al., 2025). At the population level, machine learning is used to predict antimicrobial resistance trends throughout the healthcare environment. Gradient boosting methods are shown to effectively use past resistance data, antimicrobial use patterns, and pathogen–antibiotic relationships to better predict resistance, especially in seasons of marked variability. The results indicate a need for incorporating temporal resistance patterns and treatment context, where organism–antibiotic information and previously established resistance trends prove beneficial for both patient-level prediction and population-level forecasting (Vihta et al., 2024). Other healthcare machine learning studies also demonstrate the complementary nature of ensemble learning and metaheuristic optimization. Random Forest, XGBoost, and Decision Tree were combined in a meta-ensemble for early sepsis prediction by Ansari Khoushabar and Ghafariasl, and this combination of heterogeneous learners showed potential to enhance predictive performance (Ansari Khoushabar and Ghafariasl, 2024).

Three concepts that have recently emerged in machine learning research related to infection are interpretability, calibration, and external validation. The statistical model, accompanied by explainability techniques, demonstrates good performance on the datasets. Model interpretation methods are used to identify key clinical and laboratory features that are important for predicting the risk of infection, thereby enhancing transparency in the risk assessment process. The studies highlight the importance of both feature selection and validation, as well as explainability when integrated into clinical prediction workflows (Lan et al., 2026). Multimodal approaches have also expanded the scope of AMR prediction beyond structured clinical and surveillance data. Data fusion effectively improves the performance of single-modality models, and the integration of static variables, time-series data, and clinical text within the same deep learning framework has proven to be effective. However, the predictive performance remains moderate, and the effects of class imbalance are notable, indicating a need for further efforts in multimodal modeling (Hardan et al., 2024).

Genomic and sequence-based approaches are also enhancements to AMR prediction, incorporating molecular-level information. Machine learning models that rely on features derived from sequences can be used for probabilistic antibiogram prediction, but their use in clinical practice is limited (Topcu and Akçapınar Sezer, 2026). Advances in genomic modeling highlight the need to represent and eliminate features to enhance generalizability and computational efficiency (Gerada et al., 2026). Gene copy-number profiles (CNPs), another alternative genomic feature, further enhance robustness and performance across a wide range of bacterial lineages (Fistarol et al., 2025).

In general, previous machine learning research indicates that AMR and antibiotic susceptibility prediction can be aided by identifying the organism, its resistance history, previous exposure to antimicrobials, clinical features, and multimodal or genomic data. However, the existing evidence primarily focuses on adults, particularly in ICUs, bloodstream infections, and surveillance data or genomic modeling. The prediction of pediatric susceptibility, using integrated clinical and nasopharyngeal antibiogram-linked data, is still not well explored and requires high-performance, interpretable, and efficient feature selection methods.

### 2.2 Feature selection and redundancy-aware predictor selection in biomedical machine learning

In biomedical machine learning, the dimensionality, noisy, and correlated nature of biomedical data makes feature selection vital. It enhances the robustness of the model, reduces overfitting, simplifies model interpretation, and supports clinically relevant predictions. Multi-step and ensemble-based feature selection strategies that incorporate statistical screening, machine learning ranking, stability assessment, and explainability are increasingly highlighted in recent studies. These methods reduce the number of features without compromising accuracy or coherence stemming from different selection methods (Battistella et al., 2024; Maruotto et al., 2025).

In infection-related modeling, feature selection can be commonly integrated into complex machine learning pipelines that employ class balancing, optimization, and feature-ranking. Such studies indicate that improved prediction is achieved with optimal feature selection in heterogeneous clinical and physiological datasets, although most of these papers focus on adult disease scenarios (Siri et al., 2025; Cai et al., 2025). Studies focusing on respiratory and lung-related features also underscore the value of hybrid and multi-stage feature selection methods, which combine statistical and machine learning techniques with an explainable modeling approach. These strategies enhance both robustness and interpretability in predicting diseases based on multimodal clinical and imaging data (Wu et al., 2026; Zheng et al., 2025).

The experimental results demonstrate that various families of feature selection techniques, such as filter, wrapper, and embedded methods, yield different outcomes when compared. Generally, ensemble-based or wrapper-based approaches identify fewer non-redundant and more informative predictors, leading to better predictive performance (Kendall et al., 2025; Kaliappan et al., 2024; Cai et al., 2025). Previous studies also reinforce the need for optimal feature representation and reduction methods in high-dimensional biomedical applications. Some feature selection techniques are dynamically or statistically driven, providing optimal flexibility, efficiency, and predictive accuracy in clinical and radiomics applications (Parmar et al., 2015; Lu et al., 2022; Han et al., 2021; Ansari et al., 2026).

All these studies indicate that the future of biomedical feature selection is moving towards multi-step, ensemble, and redundancy-conscious algorithms, which can enhance robustness, interpretability, and predictive accuracy. Nevertheless, most methods are aimed at a general clinical performance or disease environments in adults and are not designed to predict antibiotic susceptibility in pediatrics. This underscores the necessity for a framework specific to pediatrics that incorporates clinical, bacterial, antibiotic, laboratory, and nasopharyngeal antibiogram-linked data while considering feature selection disagreement and redundancy. The multi-method approach to predictor selection is a consensus-weighted method integrating model-based importance, statistical tests, recursive selection, and redundancy control, providing a structured approach to selecting compact and clinically interpretable predictors.

### 2.3 Gaps and the need for our framework

While recent machine learning research has focused on predicting antibiotic effectiveness, antibiotic stewardship, pneumonia prognosis, and risk prediction of respiratory infections in children, challenges remain regarding the classification of antibiotic susceptibility, modeling interactions between organisms and antibiotics, stable feature selection, and generating interpretable decision support. The key limitations of the reviewed studies, the research gaps, and how the proposed framework addresses these gaps are summarized in Table 1.

**Table 1:** Gap analysis of studies relevant to pediatric antibiotic susceptibility.

Reference	Existing limitations in literature	Identified gap	How our framework addresses it
Yang et al. (2025)	Predicts antibiotic effectiveness by using ensemble ML with moderate performance and without microbial/resistance-specific data	Only partial involvement of pathogen-specific/antibiotic-specific predictors	Framework incorporates clinical, bacterial, and antibiotic data and outcomes supported by the antibiogram for more accurate predictions
Radakovich et al. (2025)	Predicts antibiotic need only with XGBoost on a small dataset without multi-modal inputs and without a focus on susceptibility prediction	Antibiotic susceptibility for a specific microorganism is directly modeled in the framework using integrated clinical and microbiological data	The antibiotic susceptibility of a given microorganism is directly modeled in the framework based on clinical and microbiological data
Chen et al. (2025)	Mortality prediction (moderate performance, only prognosis) using Boruta + ML (machine learning)	Failure to consider the susceptibility prediction of antibiotics	New focus on antibiotic resistance/susceptibility prediction with structured clinical data in the framework
Zhu et al. (2025)	LASSO + ML (machine learning) model for treatment response, but single-center and without microbiological data	There is a need for models that are interpretable and include clinical and microbial predictors.	Framework includes bacterial and antibiotic variables, interpretable feature selection, and SHAP. Learning, statistical validation, and explainability with the SHAP tool.
Zhao et al. (2025)	Ensemble mortality model is accurate but complicated and lacks external validation	The need for compact, stable, and interpretable feature subsets.	Using consensus FS, the framework chooses sets of predictor variables with minimum variance.
Jiang et al. (2025)	XGBoost + SHAP model has not been externally validated, and there is no feature stability analysis.	Need for strong and consistent feature selection, beyond single model importance, and the importance of having robust and stable feature selection, beyond single model importance.	Multi-agent feature selection with consensus weighting is employed to enhance stability in the framework.
Yang et al. (2026)	Stacking ensemble predicts resistance but requires expensive biomarkers and is limited in external validation.	There is need for a lightweight, clinically relevant prediction models.	Stable, predictable predictors are selected for creating compact, deployable models in the framework.

As presented in the table, previous work has yielded valuable evidence regarding the prediction of respiratory infections, support for antibiotic use decision-making, ensemble learning, and interpretation via SHAP. Many studies, however, are limited to prognosis instead of susceptibility, are based on single-center data, lack microbiological or resistance-specific variables, or fail to assess feature selection stability. The proposed framework overcomes these shortcomings by combining clinical, bacterial, antibiotic, and antibiogram data with consensus-weighted multi-agent feature selection, redundancy penalization, ensemble modeling, statistical validation, and explainability via the SHAP approach.

## 3 Materials and methods

### 3.1 Study design and data description

This was a retrospective machine learning study based on a secondary analysis of a publicly available and anonymized dataset of clinical, microbiological, laboratory, and antibiogram data originally collected from pediatric participants. For the present analysis, no additional clinical or biological measures were obtained, and no new participants were recruited. The classification of antibiotic susceptibility was regarded as a prediction task and was binary (encoded as 1 for Sensitive and 0 for Resistant). A Patient–Bacterium–Antibiotic susceptibility record served as the unit of analysis. Analytical records were created for each bacterium identified in the NPA antibiogram and its susceptibility to one of the tested antibiotics, associated with the clinical and contextual data of the corresponding child. Susceptibility results might be provided for more than one organism–antibiotic combination for an individual child, and for the same child, more than one record of susceptibility might be provided for one organism–antibiotic combination; thus, the number of patient–bacterium–antibiotic records was greater than the number of children. The patient-level clinical dataset was merged with the NPA antibiogram dataset by patient identifier before discarding this code prior to model development.

There were 801 children in the source cohort. The clinical and antibiogram data were then integrated, intermediate and incomplete susceptibility results were excluded, and the results were classified during the construction of the modeling dataset, which resulted in 3,663 patient–bacterium–antibiogram records, of which 1,828 were Sensitive and 1,835 were Resistant. After eliminating non-binary and intermediate susceptibility results and equalizing susceptibility classes, a final modeling dataset of 3,663 patient–bacterium–antibiotic records was generated for the construction and evaluation of the model. Finally, the dataset was partitioned into training and held-out test sets before being preprocessed, feature-selected, and modeled.

This study did not directly evaluate the incremental predictive value of antibiotic identity, bacterial organism, clinical, inflammatory, anthropometric, environmental, and healthcare-related variables. While these variables were identified as being selected by the feature selection framework and found to contribute to the prediction in the SHAP analysis, this does not necessarily indicate better predictive performance compared to a baseline model consisting only of the organism and antibiotic. The taxonomy of features is presented in Table 2.

**Table 2:** Taxonomy of the 64 candidate predictors.

Feature domain	*n*	Representative variables
Antibiogram-linked/microbiological	2	Bacterial organism; tested antibiotic
Socio-environmental and exposure-related	7	Persons in household; siblings; rooms in house; smokers at home; tuberculosis contact; breastfeeding; medical insurance
Clinical – demographics / anthropometry	4	Age; sex; weight; height
Clinical – medical history and prophylaxis	10	Previous respiratory admission; prematurity; vaccination status; vaccine doses; vitamin A; recent antibiotic use; known asthma; chronic condition
Clinical – presenting symptoms	7	Fever; duration of fever; vomiting; diarrhea; cough; rhinorrhea; duration of pain
Clinical – vital signs	4	SaO₂; axillary temperature; respiratory rate; heart rate
Clinical – examination signs	13	Pneumonia criteria; sleepiness; paleness; consciousness disturbance; dehydration; restlessness; cyanosis; nasal flaring; stridor; rhonchi; crackles; wheezing; hypoventilation
Clinical – laboratory	2	C-reactive protein; procalcitonin
Clinical – radiology	1	Chest X-ray finding
Clinical – in-hospital management	11	NPA procedure; oxygen therapy; bronchodilators; corticosteroids; in-hospital anti-biotherapy; empiric antibiotic indicators
Clinical – in-hospital course	3	Length of hospital stay; ICU transfer; ICU length of stay
Total	64	—

### 3.2 Data preprocessing pipeline

The clinical and nasopharyngeal aspirate (NPA) antibiogram datasets were cleaned and harmonized first and then combined to create the final analytical dataset. Some variables were excluded from the analytical dataset during construction, such as those with too many missing values, those with little analytical relevance, and those with obvious potential for outcome leakage, including selected laboratory, diagnostic, and outcome-related variables. After the train–test split, missing-value handling was performed on the training partition. For applicable binary variables, categorical variables were set to clinically appropriate default categories, count variables were set to zero, and continuous variables were imputed with the mean values of the applicable variables in the training data. The imputation parameters were then fitted for the held-out test partition.

These categorical data were then transformed into numerical representations, following the implemented binary and ordinal coding schemes. If encoding had to be derived from the data, the encoding procedure was fitted to the training partition and then applied, without modification, to the test partition. Categorical variables were re-coded as binary variables, while ordered categorical variables were represented by ordered numerical variables corresponding to the levels of the categorical variable. All continuous and count variables were numerically retained after applying the missing value procedures listed above. The identities of the antibiotics and bacteria were considered high cardinality categorical variables and were encoded using the applied hybrid frequency–label encoding. Each encoding required observations from the data, so the mapping for each encoding was estimated using the training partition and then applied directly to the held-out test partition. Only Sensitive and Resistant susceptibility results were included in the antibiogram data; those with intermediate or incomplete susceptibility were excluded. The final analytical dataset consisted of 3,663 records, generated by random subsampling during the construction of the final dataset (as described in Section 3.1). Class equalization was performed through random subsampling. The entire data preparation procedure is summarized in Figure 1.

**Figure 1:**
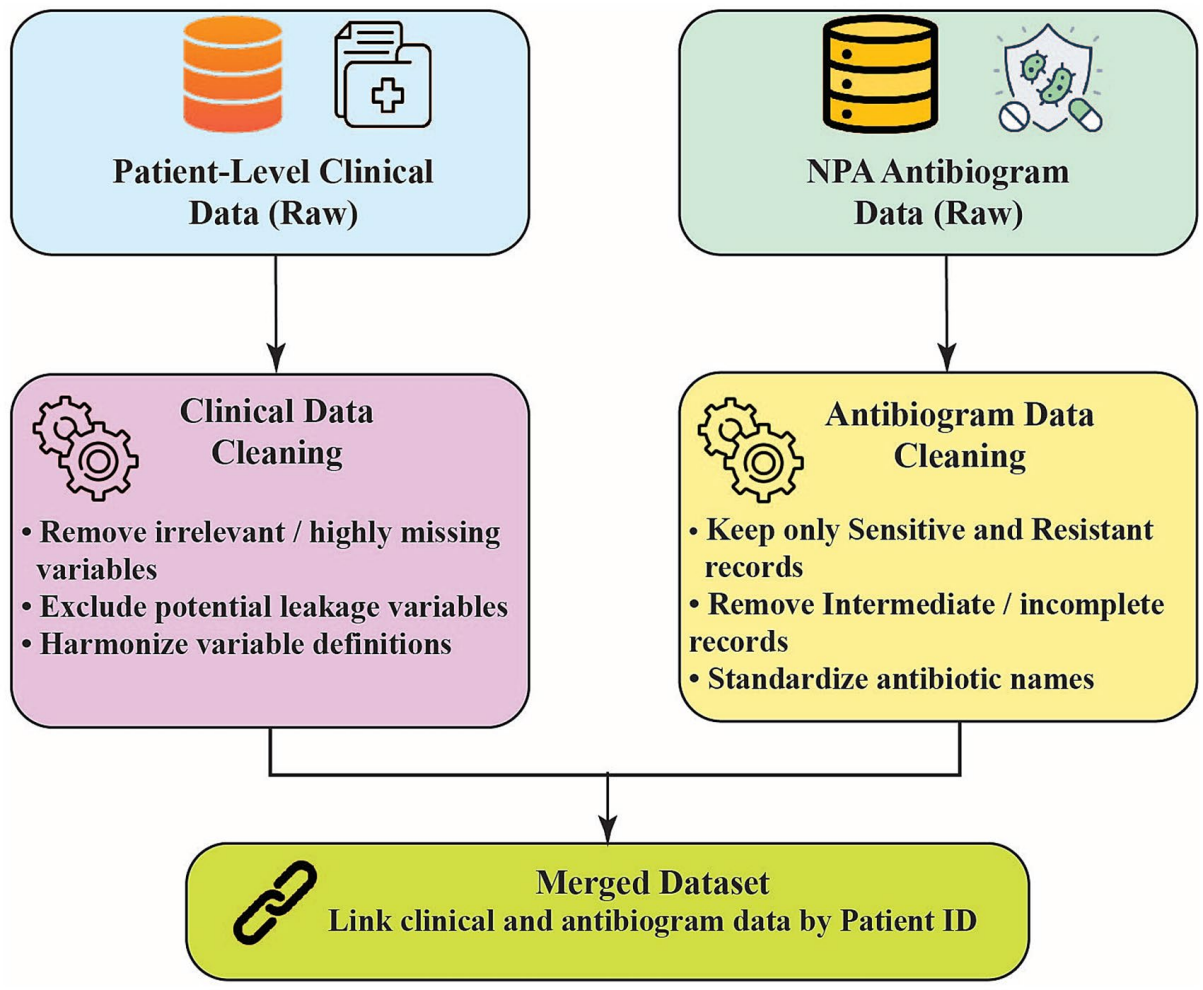
Data preparation and preprocessing workflow.

Antibiotic and bacterial names were standardized by lowercasing, removing special characters, and harmonizing inconsistent labels. The clinical and NPA antibiogram datasets were then integrated using the patient identifier to generate patient–bacterium–antibiotic records. Class equalization was performed through random subsampling, yielding 3,663 records for subsequent train–test partitioning.

After the train–test split, missing-value imputation and data-dependent encoding were fitted using only the training partition and then applied unchanged to the held-out test partition. Binary and categorical variables used appropriate default categories; count variables used zero where applicable, and continuous variables were imputed using training set means. Binary, ordinal, and hybrid frequency–label encoding were subsequently applied, with data-derived mappings estimated from the training data. The preprocessed training set was used for feature selection and model development, whereas the identically transformed test set was reserved exclusively for final evaluation. The preprocessing workflow is summarized in Figure 1.

### 3.3 Data splitting

The final modeling dataset of 3,663 patient–bacterium–antibiotic records was partitioned at the record level using an 80:20 stratified train–test split to preserve the susceptibility class distribution, resulting in 2,930 training records and 733 held-out test records. The patient identifier was used only for the integration of the clinical and antibiogram datasets and was removed before model development; therefore, it was not included as a predictor. The implemented record-level partitioning did not explicitly enforce separation of all records belonging to the same patient across the training and test sets.

After partitioning, all subsequent data-dependent preprocessing, feature selection, cross-validation, development of classifiers, and model selection were conducted on the training partition. All of these procedures were carried out without the held-out test partition, which was used only for final model evaluation.

### 3.4 Proposed consensus-weighted multi-agent feature selection framework

The proposed framework aimed to discover relevant and minimally redundant predictors for antibiotic susceptibility in children by combining different perspectives of feature selection. To achieve this, six different feature selection agents were used: Random Forest importance, Gradient Boosting importance, ANOVA F-test, Mutual Information, recursive feature elimination (RFE) with Random Forest, and recursive feature elimination (RFE) with Gradient Boosting. These agents can be classified as embedded, filter-based, or wrapper-based.

Each agent assessed and ranked potential predictors based on the underlying system’s relevancy criteria. The scores were not directly comparable and were normalized before integration. The normalized scores were then combined using a consensus weighted fusion module to determine the overall importance of each feature. The vote-strength metric was also calculated, representing the extent to which each feature was chosen by all of the agents.

A correlation-based redundancy penalty was added to the final scoring method to avoid highly correlated predictors. Consequently, features supported by more than one agent but with less redundancy were prioritized. A top 20 feature subset, which is the most informative with minimal redundancy, was selected from the finalized ranking to develop and ensemble the downstream machine learning model. The proposed overall architecture is shown in Figure 2.

**Figure 2:**
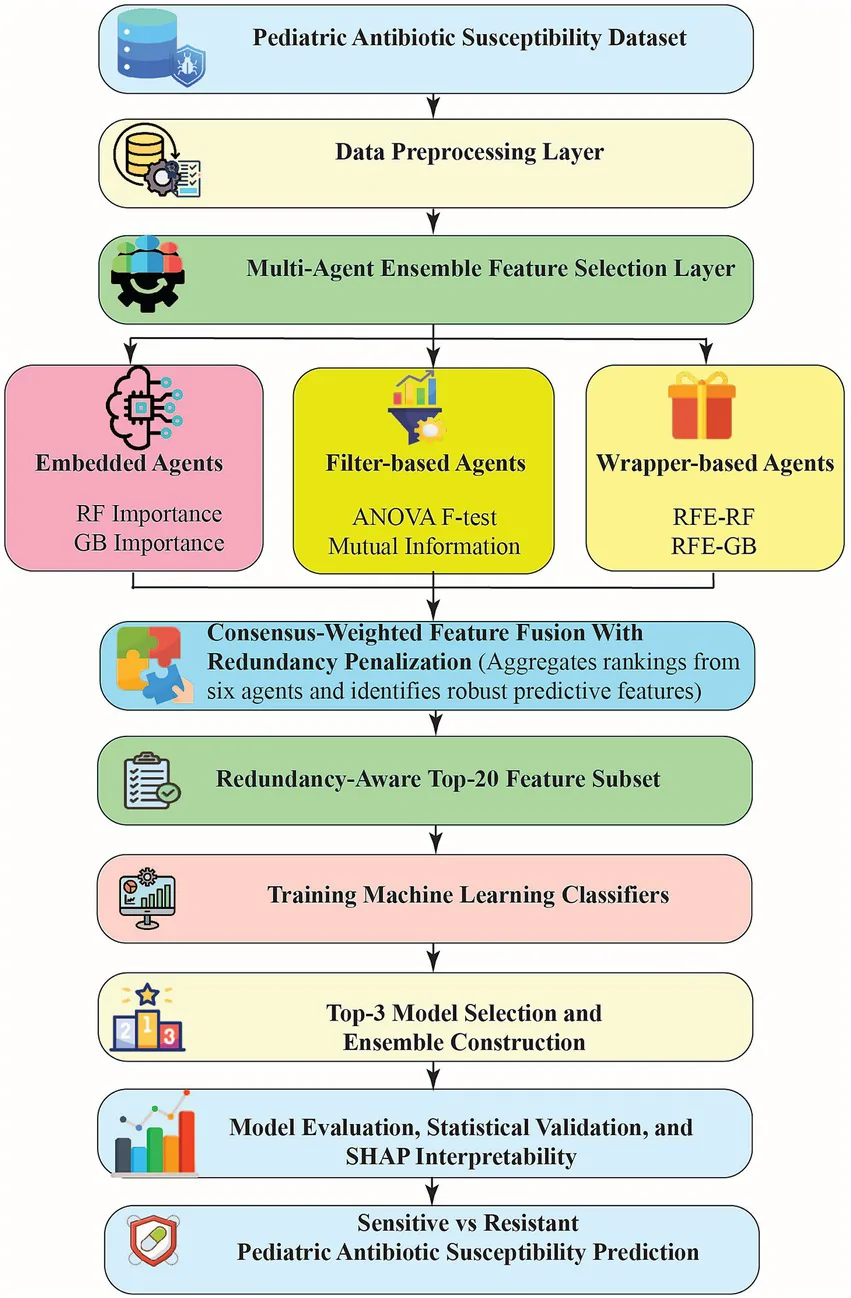
Proposed architecture of consensus-weighted multi-agent feature selection for pediatric antibiotic susceptibility prediction.

The workflow starts with antibiotic susceptibility data from the pediatrics, which is then preprocessed before a multi-agent feature selection layer is applied. Three different types of feature selection agents (embedded, filter, and wrapper) assess candidate predictors independently. They are then fused together via a consensus weighted fusion module with redundancy penalization to determine a refined top feature subset. These selected features are then fed into the machine learning models, building an ensemble predictor that is statistically validated and interpreted using the SHAP method. The end result is a binary antibiotic susceptibility result: sensitive vs. resistant.

### 3.5 Feature selection agents

The proposed framework involved the use of six complementary feature selection agents to assess predictor relevance through various methodological approaches. These agents were classified as embedded, filter-based, and wrapper-based. The embedded agents were based on Random Forest importance and Gradient Boosting importance, which estimated the importance of features according to the structure of the trees. The filter-based agents were the ANOVA F-test and Mutual Information, which checked the separation of different statistical classes and the nonlinear dependency between the agents and the target, respectively. The recursive feature elimination agents, based on Random Forest and Gradient Boosting, eliminated the least informative features based on the model’s score.

Each agent generated its own feature ranking or feature importance score. To minimize reliance on any single feature selection criterion and to provide complementary relevance patterns from a variety of statistical, information-theoretic, and model-based methods, this multi-agent design was employed. Table 3 outlines the six feature selection agents and their approach to feature selection.

**Table 3:** Multi-agent feature selection framework.

Category	Agent	Technique	Concept	Working Mechanism	Output/eole
Embedded Agents	Agent 1	Random Forest Importance	Ensemble of parallel decision trees	Constructs multiple decision trees; calculates the average Gini impurity reduction over all splits;	Features with the highest impurity reduction in the splits are more important on average
	Agent 2	Gradient Boosting Importance	Sequential error-correcting trees	Builds trees sequentially; each tree focuses on reducing variance (MSE reduction)	Features that are consistently good at reducing variance are given higher weights
Filter-Based Agents	Agent 3	ANOVA F-Test	Statistical variance comparison	Divides data into Resistant vs. Sensitive groups; calculates ratio of between-group variance to within-group variance	Larger F-test scores indicate more discriminative power.
	Agent 4	Mutual Information	Information-theoretic dependency measure	Estimates the dependency between feature and target with a nearest-neighbor-based approach	Captures non-linear relationships and assigns higher scores to informative features
Wrapper Agents	Agent 5	RFE (Random Forest)	Iterative backward elimination	Trains a Random Forest model iteratively, eliminating the least important feature at each step until the desired subset size is obtained	Optimized subset based on ensemble tree evaluation
	Agent 6	RFE (Gradient Boosting)	Iterative backward elimination	Feature selection: Removes the least accurate feature sequentially; Gradient Boosting (50 rounds, max depth = 3)	Generates an optimized subset for sequential boosting models

### 3.6 Consensus-weighted feature fusion

Six feature selection agents obtained different importance scores based on various statistical and model-based approaches, making it difficult to compare the raw scores of these agents. All agent-specific feature scores were normalized for consistency of scale before being fused, using min–max normalization. This change allowed all agent outputs to be mapped onto a common scale and prevented any single agent from exerting excessive influence over the fusion process due to differences in scores. All agent-specific scores and normalization parameters were derived exclusively from the training partition.

Normalization was performed, and a consensus score was calculated for each feature as the average of the normalized feature importances of the six agents. This score represented an overall assessment based on the multi-agent evidence, reflecting the relevance of each predictor. In addition, cross-agent agreement was quantified by vote strength. The 20 best-ranked features for each of the six feature selection agents were considered the selected features for that agent. For a candidate feature fi, vote strength *V_i_* was calculated as follows:


$$V_{i}=\frac{1}{6}\;\sum_{j=1}^{6}1(f_{i}\in T_{j})$$

where $T_{j}$ denotes the Top-20 feature set produced by feature selection agent J , and 1(.) is an indicator function that equals 1 when feature $f_{i}$ belongs to $T_{j}$ and 0 otherwise. Accordingly, $V_{i}$ ranged from 0 to 1, with larger values indicating stronger cross-agent agreement. For example, a feature included in the Top-20 sets of all six agents received $V_{i}=1.0$ , whereas a feature included by three of the six agents received $V_{i}=0.5$ . Thus, vote strength quantified how consistently a feature was retained among the highly ranked predictors across the six complementary feature selection methods.

### 3.7 Redundancy penalization and final feature ranking

A correlation-based reduction of multicollinearity and a limit on the number of overlapping predictor variables were added to the final feature ranking process. Consensus weighted fusion identifies features selected by many selection agents, but it does not directly consider inter-feature dependency. Therefore, it was decided to use an additional layer of control, redundancy analysis, to enhance feature diversity and model robustness.

Redundancy was evaluated based on the training set only, and the pairwise association measure was chosen based on the type of feature measurement. Absolute Pearson correlation was applied for continuous, count, and binary numerical variables; for binary–binary associations, it is equivalent to the Pearson phi coefficient, while for binary–continuous associations, it is the same as point-biserial correlation. For associations between explicitly ordinal variables, Spearman rank correlation was used. Multi-category nominal predictors (bacterial identity, antibiotic identity, and chest X-ray category) were not correlated with one another using Pearson correlation because they have arbitrary numerical values that do not represent meaningful metric distances. For nominal–nominal and nominal–binary associations, a bias-corrected Cramér’s V was used, while correlation ratio (η) was used for nominal–nonbinary numerical associations. The magnitude of the pairwise association was represented by $R_{\mathrm{ij}}$ , which takes on values from 0 to 1.

To determine strongly associated pairs of predictors, a threshold of $R_{\mathrm{ij}}>0.80$ was set. The rationale for this threshold was that it would restrict the amount of redundancy penalization to predictors with high overlap, while allowing reasonable penalization for associated variables contributing to the prediction. The redundancy set for each feature $f_{i}$ was then defined as.


$$\Omega_{i}=\{\;j:R_{\mathrm{ij}}>0.80,j\neq i\}$$

The redundancy penalty was subsequently calculated as


$$\Lambda (f_{i})=\{\begin{array}{c} \frac{1}{|\Omega_{i}|}\sum_{j\in \Omega_{i}}R_{\mathrm{ij}},|\Omega_{i}|>0,\\ 0,|\Omega_{i}|=0. \end{array}$$

In this way, some features were assigned a greater penalty due to strong dependency on other candidate predictors, while others received zero penalty if no other predictors showed a stronger dependence on them than the predefined threshold. A threshold sensitivity analysis with $R_{\mathrm{ij}}>0.70$ , $R_{\mathrm{ij}}>0.80$ , and $R_{\mathrm{ij}}>0.90$ was also employed to determine the robustness of the final feature ranking to the selected threshold. The final feature score was defined as a combination of consensus relevance, cross-agent vote strength, and redundancy penalization:


$$S_{i}=0.60C_{i}+0.30,V_{i}-0.10,\Lambda (f_{i}).$$

where $S_{i}$ is the final score of the features $f_{i}$ , $C_{i}$ is the consensus importance score and is obtained from the normalized outputs of the six feature selection agents, $V_{i}$ is the vote strength representing the proportion of agents that selected feature $f_{i}$ , according to the predefined selection rule, and $\Lambda (f_{i})$ is the redundancy penalty assigned to feature $f_{i}$ . The weighting scheme aimed to reflect the relative roles of the three components in the proposed framework. The consensus score received the highest weight (0.60), as the aggregate feature relevance values across the six complementary feature selection methods were the most important evidence for retaining a predictor. The strength of the vote was given a secondary weight (0.30) to support features that were consistently favored across agents. A smaller weight (0.10) was assigned to the redundancy penalty, as it was used to correct for highly redundant predictors without overpowering strong evidence of predictor relevance. Therefore, the weighting was based on aggregate cross-method relevance, with cross-agent agreement given second priority, and redundancy receiving secondary penalization. The coefficients were not interpreted as theoretically optimal values.

A Top-20 cutoff was chosen *a priori* as a parsimonious design choice to ensure that enough predictors are included in the design for modeling purposes while providing reasonable dimensionality reduction. We reduced the original 64-dimensional space to 20-dimensional predictors, resulting in a 68.75% reduction in dimensionality, which limits the number of inputs fed to the classifiers and reduces unnecessary model complexity and computational burden. Another set of 20 predictors was considered sufficiently large to retain complementary information provided by the diverse feature selection agents, but not so large that it required only a small number of key microbiological predictors. The resulting subset comprised predictors related to microbiological, physiological, inflammatory, anthropometric, environmental, radiological, and healthcare factors. The number 20 was therefore not chosen as a theoretically optimal number of predictors but rather as a pragmatic and representative feature-budget parameter. The same Top-20 criterion was uniformly applied during the agent-level voting procedure and the final stage of feature retention.

A resampling-based stability analysis was conducted to quantitatively assess the stability of the feature selection, using 20 stratified bootstrap replicas of the training data. Sampling of observations was done with replacement, and the distribution of classes was maintained in each repeat. The entire six-agent feature selection process was re-fitted, incorporating consensus scoring, vote-strength computation, redundancy penalty, and final Top-20 ranking. The measures of subset stability included pairwise Top-20 overlap, Jaccard similarity, and the chance-corrected Kuncheva index. The stability of the entire 64-feature rankings was determined by pairwise Spearman rank correlation, while feature-specific stability was calculated as the percentage of bootstrap repetitions in which a particular predictor appeared in the Top-20 subset. The held-out test set was not used in the stability analysis.

### 3.8 Machine learning classifier development

The features were selected through a consensus-weighted multi-agent feature selection procedure aimed at identifying the most relevant, consensus-supported, and non-redundant predictors, whose resampling stability was subsequently quantified using the procedure described above. The selected features represent major factors affecting the susceptibility of children to antibiotics from microbiological, physiological, and environmental perspectives. Resistance patterns have a strong association with prior antibiotic exposure and the identity of the bacteria. Physiological parameters (respiratory rate, heart rate, oxygen saturation, temperature, white blood cell count) and inflammatory markers (C-reactive protein, procalcitonin) are significant for diagnosing clinical severity. Underlying immune competence is influenced by developmental and health status factors such as height, weight, and vaccination history. Household size and housing conditions are environmental factors that can help understand exposure and risk of transmission. Previous hospitalization and symptom duration are included in clinical history, which measures healthcare exposure and the progression of infection; oxygen therapy indicates acute disease severity. Additional demographic information is provided by gender.

Top 20 feature subsets were reduced and used as input for the development of classifiers. A variety of classifiers was implemented using the selected feature subset to assess its predictive power, encompassing a wide range of learning paradigms such as linear, probabilistic, instance-based, margin-based, tree-based, and ensemble classifiers. The classifiers used in the evaluations included Logistic Regression (LR), Linear Discriminant Analysis (LDA), Stochastic Gradient Descent (SGD) classifier, Naïve Bayes (NB), k-Nearest Neighbors (KNN), Support Vector Classifier (SVC), Nu-Support Vector Classifier (NuSVC), Decision Tree (DT), Random Forest (RF), Extra Trees (ET), Extreme Gradient Boosting (XGBoost), and Light Gradient Boosting Machine (LightGBM). The best 20 feature subset obtained from the training set was then tested using stratified five-fold cross-validation of the classifiers. Feature selection was not performed independently within each fold of the cross-validation; thus, the cross-validation estimates represent performance conditioned on the selected feature representation and do not correspond to fully nested estimates of the complete feature selection and modeling pipeline. The held-out test partition was not used in feature selection or cross-validation and was reserved for final model evaluation.

### 3.9 Genetic algorithm-based candidate model selection

The Genetic Algorithm, combined with the Decision Tree Classifier, yielded the best results with 100% diagnostic accuracy on their test set compared to the standard K-Means clustering and Support Vector Machines, which were less effective in predicting the exact stage of lung cancer (Younis et al., 2025). A genetic algorithm (GA) was employed as a heuristic search strategy to identify a three-classifier ensemble from the 12-candidate machine learning models. Each candidate solution consisted of three different classifiers. The GA was run for 15 generations, starting with 20 potential combinations and applying tournament selection with a tournament size of 3, a mutation rate of 0.30, and elitism that preserved the two best-fitting solutions for each generation. To ensure reproducibility, a fixed random seed of 42 was used. At the crossover stage, unique classifiers from two parent solutions were combined, and three classifiers were selected to create an offspring solution. One classifier was replaced with a simpler classifier that was not included in the set of candidate classifiers. The fitness was calculated as the ROC-AUC, derived from the equally averaged predicted probabilities of the three constituent classifiers.

Because the present search space contained only $(\begin{array}{c} 12\\ 3 \end{array})=220$ possible three-model combinations, exhaustive enumeration was computationally feasible. In this work, therefore, it was unnecessary to minimize computation costs in the GA; instead, the results were evaluated as a heuristic ensemble selection approach that can be extended to larger pools of classifiers and ensemble sizes. All 220 combinations were also exhaustively evaluated as a deterministic benchmark. The GA and exhaustive search both identified the same three-classifier combination, indicating that the GA found the best combination within the searched space (highest ROC-AUC). Thus, the result from the exhaustive search was used to validate the GA result.

### 3.10 Model assessment metrics and justification

Multiple classification and discrimination measures were used to assess model performance. The task was designed in a binary classification format where the positive class represented sensitive records and the negative class represented resistant records, following the outcome encoding applied to the task. Recent papers highlight the need for multiple machine learning performance metrics to reliably assess models for lung cancer predictions. Metrics such as accuracy, precision, recall, F1-score, and ROC–AUC provide a detailed evaluation of models and allow for fair comparisons among them. Optimized evaluation strategies also enhance prediction accuracy and clinical utility in decision-making (Ansari et al., 2026; Husaini, 2026). The following metrics were evaluated: accuracy, precision, recall/sensitivity, specificity, F1-score, ROC-AUC, Matthews correlation coefficient (MCC), log loss, and confusion matrix analysis. Accuracy measured overall correctness, while resistance prediction performance was assessed using precision and recall. Specificity evaluated the ability to correctly identify sensitive records. The F1-score achieved a balance between precision and recall, and the MCC offered a comprehensive measure of balance by considering all elements of the confusion matrix. Threshold-independent discriminative ability was assessed using ROC-AUC, while probability calibration was measured by log loss.

Confusion matrix analysis was conducted based on the implemented outcome coding, with sensitive records coded as positive (true positive) and resistant records coded as negative (true negative). In this case, true positives were those records correctly identified as sensitive, true negatives were those correctly identified as resistant, false negatives were those incorrectly identified as resistant, and false positives were those incorrectly identified as sensitive. Class-specific error patterns were explored alongside overall error measures to aid in the clinical interpretation of model performance.

### 3.11 Statistical validation

A statistical validation was conducted to test the model’s generalization, discrimination, consistency in classification, and uncertainty of performance estimates. The validation process comprised paired model comparisons, held-out test assessments, stratified cross-validation, and confidence interval calculations. Recent studies have emphasized the importance of rigorous statistical validation in machine learning-based cancer prediction. This enhances the reliability of the models and aids in their performance evaluation through methods such as cross-validation, confidence intervals, and hypothesis testing. The integration of multimodal data with statistically validated evaluation frameworks can facilitate accurate and clinically meaningful risk stratification (Singh et al., 2025; Yagci et al., 2026). Stratified K-fold cross-validation (stratKFCV) was performed to assess model stability, utilizing training data to fit the model and validation data to estimate its performance using consistent evaluation metrics. Confusion matrix analysis, accuracy, sensitivity, specificity, precision, F1-score, ROC-AUC, Matthews correlation coefficient (MCC), and log loss were used to evaluate the final selected model over a held-out test set. DeLong’s test was used to assess whether there was a difference between the ROC-AUC results of the ensemble and the baseline classifier, while a McNemar test was performed to compare the paired errors by examining the discordant predictions of the classifiers. Confidence intervals were used to estimate the uncertainty of the results. All bootstrap resampled confidence intervals and Wilson score intervals were computed for sensitivity and specificity, as well as for the ROC-AUC and other performance measures.

### 3.12 SHAP-based explainability analysis

Selected predictors were analyzed using SHAP to understand how each predictor contributes to the prediction of antibiotic susceptibility. Model training was performed using the final top features obtained from the proposed feature selection framework, followed by analysis after model training.

TreeSHAP was used to generate feature attribution values for tree-based models, such as LightGBM, XGBoost, and Decision Tree. The evaluation dataset was used to calculate SHAP values, which measure the contribution of each feature in terms of magnitude and direction. The mean absolute SHAP values were used to summarize global feature importance. To interpret at the ensemble level, model-specific SHAP values were combined using the same weighting as in the ensemble model, resulting in a single feature importance profile. This aggregation was solely for interpretability and not for model training. To evaluate feature influence and ranking, summary (beeswarm) and bar plots of SHAP values were created for visualizing global interpretability. To analyze the effects of feature values on the probabilities of prediction resistance, dependence plots were appended to the key predictors. Selected cases were analyzed for local interpretability by calculating instance-level SHAP values, which show the contributions of each feature to each prediction. All SHAP analyses were conducted as *post hoc* analyses for model transparency and not as causal relationships.

## 4 Results and discussion

### 4.1 Dataset characteristics and class distribution

The study included clinical and NPA antibiogram information from 801 pediatric patients. Following the integration of the clinical and antibiogram datasets, exclusion of intermediate and incomplete susceptibility outcomes, and class equalization, the final modeling dataset comprised 3,663 Patient–Bacterium–Antibiotic records, including 1,828 Sensitive and 1,835 Resistant records. The modeling feature space contained 64 candidate predictors, comprising clinical and encoded microbiological and antibiotic-related variables. Sensitive outcomes were encoded as 1 and Resistant outcomes as 0. The finalized dataset was partitioned using an 80:20 stratified train–test split, resulting in 2,930 records for model development and 733 records for held-out evaluation. Subsequent data-dependent preprocessing and feature selection procedures were performed using the training partition, while the held-out test partition was reserved for final model evaluation.

### 4.2 Multi-agent feature selection results

The six feature selection agents detected complementary patterns of predictors in the microbiological and antibiotic variables, as well as patient-level clinical variables. Both embedded agents using Random Forest and Gradient Boosting consistently emphasized encoded antibiotic identity, encoded bacterial organism, and heart rate, highlighting the critical importance of organism–antibiotic interactions and clinical status. Antibiotic-related variables were found to be dominant predictors by the filter-based agents, while patient-level variables like age, length of stay, and number of siblings were identified as dominant predictors by the wrapper-based agents. The differences among agents validate the need for a multi-agent feature selection strategy. The tree-based agents learned the importance of the models, the filter agents identified the statistically discriminative predictors, and the wrapper agents selected features by iteratively removing them to improve model performance. The heterogeneous outputs were then fused using consensus weighted fusion and redundancy penalization of the outputs. All feature selection results were derived exclusively from the training partition.

### 4.3 Consensus-weighted top-20 feature subset

The consensus-weighted multi-agent framework identified a final subset of 20 predictors from the original 64-feature space. The features included the usual antibiotic-associated, microbiological, physiological, inflammatory, anthropometric, environmental, radiological, and hospital admission-related features, which were applied across all models used. Encoded antibiotic and bacterial variables were key predictors, highlighting the significance of the interaction between organism and antibiotic. Physiological and inflammatory parameters, including heart rate, respiratory rate, oxygen saturation, temperature, C-reactive protein, and procalcitonin, reflected acute clinical conditions. Patient-level context was obtained from anthropometric indicators, including height, weight, and age, while environmental exposure was represented by household variables such as the number of persons, rooms, and siblings. Other variables provided information about the course of the disease and its management, including the duration of fever, duration of pain, hospital stay, time in ICUs, chest X-ray results, and oxygen treatment. The selected features suggest that antibiotic susceptibility is influenced by a combination of microbiological, clinical severity, patient factors, environmental factors, and healthcare factors. The resulting 20-feature panel represented a 68.75% reduction from the original 64 candidate predictors while preserving variables from multiple complementary clinical and microbiological domains. This reduced representation was subsequently used consistently across all classifiers.

#### 4.3.1 Sensitivity analysis of the selected feature subset

Association thresholds of 0.70, 0.80, and 0.90 were examined, with 0.80 as the main threshold. In comparing the two configurations, the 0.70 configuration had all 20 features (Jaccard similarity = 1.000), while the 0.90 configuration had 19 of the 20 features (Jaccard similarity = 0.905). The Spearman correlations of the feature rankings were also high, with values of 0.983 and 0.984 for the 0.70 and 0.90 configurations, respectively, when compared with the 0.80 configuration. Furthermore, comparison with a pure Pearson-only redundancy implementation confirmed that the mixed-type association procedure did not change the features in the Top-20 final feature subset, except for the ordering of features. The results confirm that the feature subset was stable across a moderate range of redundancy thresholds and across measurements that are sensitive to the type of measurement or estimation of the pairwise redundancy.

#### 4.3.2 Resampling-based feature selection stability

The resampling analysis showed consistent feature selection across 20 stratified bootstrap repetitions of the training data. The Top-20 subsets had an average of 17.62 features (mean ± SD) in common across the 190 pairwise comparisons. The mean pairwise Jaccard similarity was 0.7890, with a standard deviation of 0.0603; the chance-corrected Kuncheva stability index was 0.8266, with a standard deviation of 0.0542; and the mean Spearman rank correlation across the entire 64-feature rankings was 0.7854, with a standard deviation of 0.0474. Compared to the Top-20 subset based on the entire training dataset, the bootstrap repetitions retained an average of 17.35 ± 0.67 features. At the feature level, 13 out of 20 predictors in the reference Top-20 subset were selected in every bootstrap repetition, 16 were selected in 90% of repetitions, and 17 were selected in 80% of repetitions. These results suggest overall resampling stability, with more variability among predictors toward the lower end of the Top-20 ranking.

### 4.4 Base machine learning classifier performance

On the consensus-selected top 20 features, the performance of the 12 machine learning classifiers was assessed. In general, the boosting-based models and tree-based models outperformed the linear, probabilistic, and instance-based models. Among the individual models, LightGBM achieved the best performance with an accuracy of 0.8718, precision of 0.8656, recall of 0.8798, specificity of 0.8638, F1-score of 0.8726, and ROC-AUC of 0.9498, followed by XGBoost with an accuracy of 0.8636, precision of 0.8500, recall of 0.8825, specificity of 0.8447, F1-score of 0.8660, and ROC-AUC of 0.9445. The moderate discriminative ability of the Decision Tree and Random Forest classifiers, with ROC–AUC scores of 0.9098 and 0.9109, respectively, along with the comparatively poor performance of Naïve Bayes, SGD, and LDA, underscores the importance of nonlinear modeling. These findings indicate that boosting-based models better represent the complex relationships between the antibiotic, microbiological, and clinical variables, as shown in Table 4. Figure 3 displays the core metric comparisons, while the additional metrics are summarized in Figure 4. For the implemented encoding (sensitive = 1), the results for recall and specificity are both clinically relevant and indicate sensitivity class detection and the correct identification of resistant cases, respectively.

**Table 4:** Performance metrics of various machine learning classifiers.

Model	Accuracy	Precision	Recall	F1-Score	Specificity	Log Loss	Erro Rate	MCC	ROC- AUC
Random Forest (RF)	0.8158	0.7770	0.8852	0.8276	0.7466	0.3822	0.1842	0.6379	0.9109
Decision Tree (DT)	0.8199	0.7812	0.8880	0.8312	0.7520	0.7196	0.1801	0.6459	0.9098
Nu Support Vector (NuSVC)	0.7885	0.7425	0.8825	0.8065	0.6948	0.4785	0.2115	0.5877	0.8457
Extreme Gradient Boosting (XGB)	0.8636	0.8500	0.8825	0.8660	0.8447	0.3039	0.1364	0.7277	0.9445
Naïve Bayes (NB)	0.7613	0.7175	0.8607	0.7826	0.6621	0.6512	0.2387	0.5333	0.8021
K-Nearest Neighbors (KNN)	0.7817	0.7814	0.7814	0.7814	0.7820	1.9002	0.2183	0.5634	0.8433
Stochastic Gradient Descent (SGD)	0.7503	0.7316	0.7896	0.7595	0.7112	0.8250	0.2497	0.5023	0.7822
Extra Trees (ET)	0.7981	0.7444	0.9071	0.8177	0.6894	0.4522	0.2019	0.6110	0.8841
Logistic Regression (LR)	0.7817	0.7395	0.8689	0.7990	0.6948	0.5104	0.2183	0.5723	0.8174
LightGBM (LGBM)	0.8718	0.8656	0.8798	0.8726	0.8638	0.2981	0.1282	0.7436	0.9498
Linear Discriminant Analysis (LDA)	0.7763	0.7265	0.8852	0.7980	0.6676	0.5197	0.2237	0.5663	0.8149
Support Vector Classifier (SVC)	0.7940	0.7483	0.8852	0.8110	0.7030	0.4742	0.2060	0.5982	0.8483

**Figure 3:**
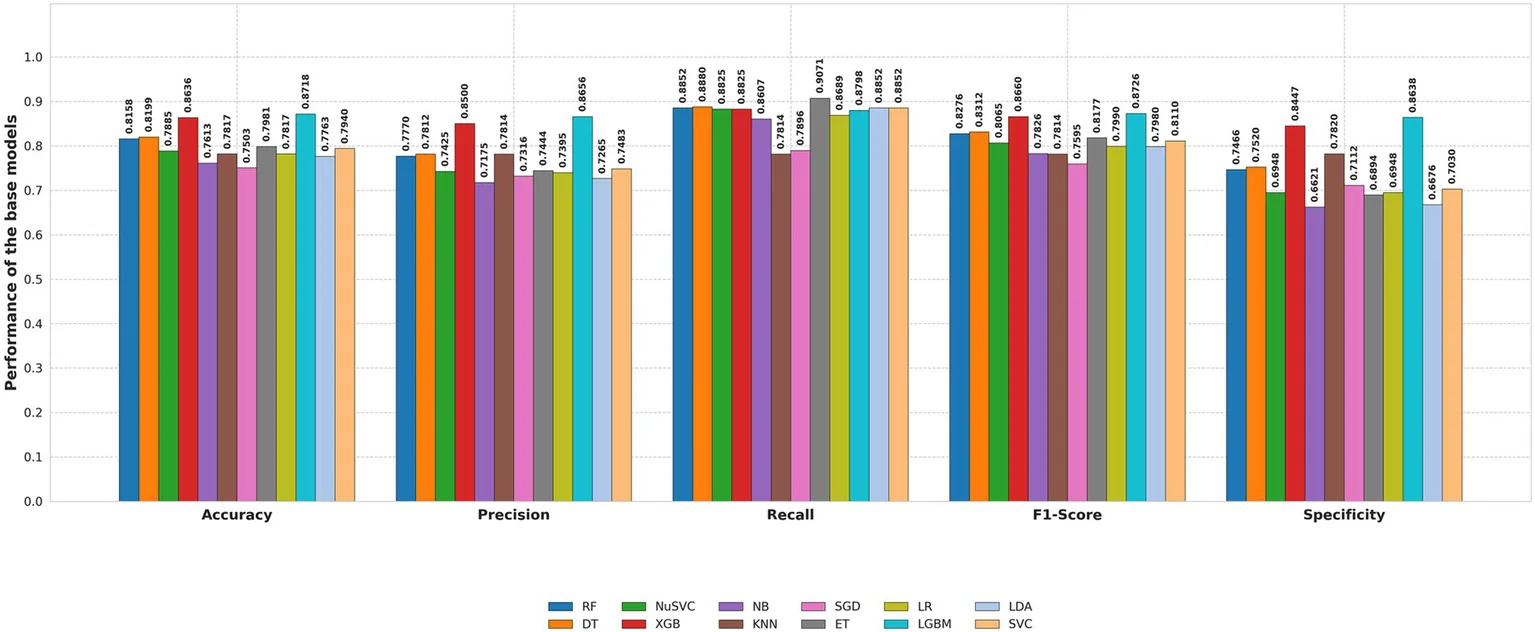
Performance of baseline machine learning models. The figure compares twelve individual classifiers across accuracy, precision, recall, F1-score, and specificity.

**Figure 4:**
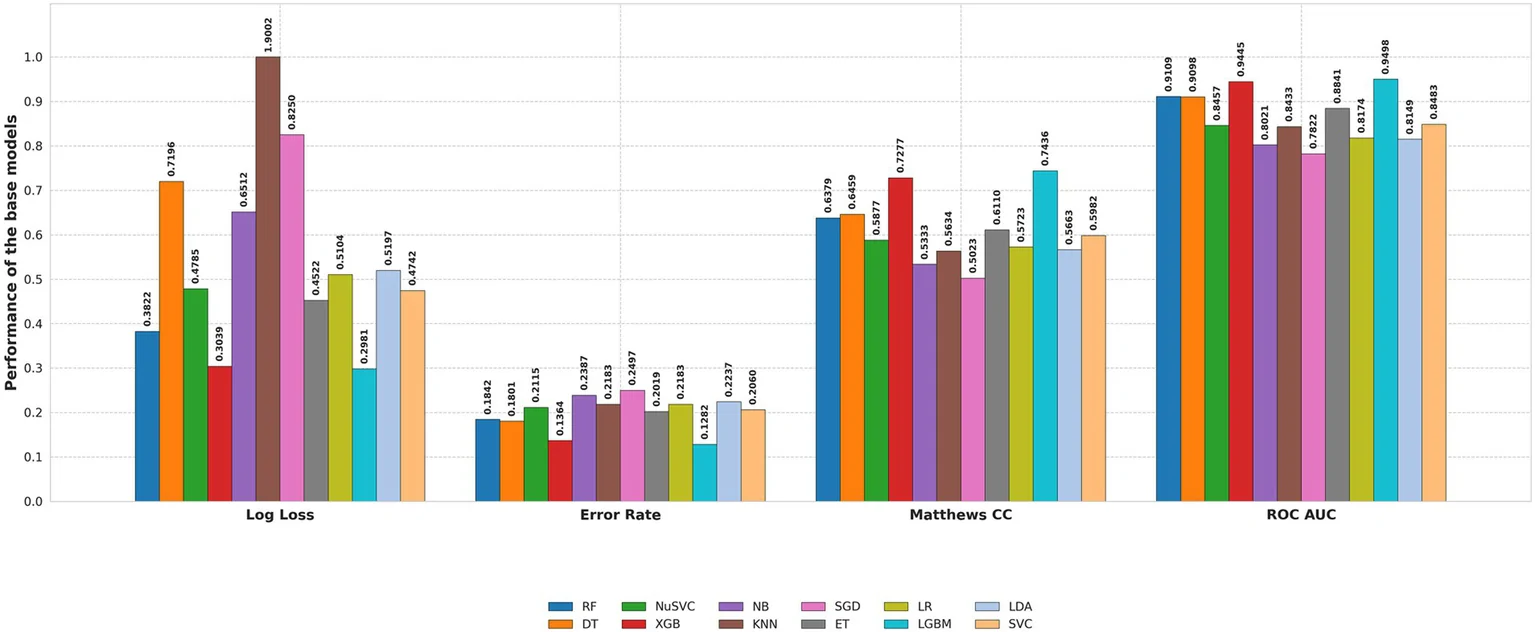
Performance of baseline machine learning models. The figure compares 12 individual classifiers across log loss, error rate, Matthews correlation coefficient, and ROC-AUC.

### 4.5 Genetic algorithm-based candidate model selection

An ensemble of exactly three different classifiers was selected from the 12 candidate classifiers using a heuristic approach: Genetic Algorithm (GA). The parameters for the GA were set as follows: population size of 20, 15 generations, a mutation probability of 0.30, tournament selection with a tournament size of 3, elitism with two best solutions per generation, and a fixed random seed of 42. Crossover selected the special classifiers from two solutions and drew three classifiers to create an offspring, while mutation chose a classifier and swapped it for another that was not already selected. The fitness was the ROC-AUC calculated using the equally weighted average of probabilities predicted by the three classifiers.

The GA found that LightGBM, XGBoost, and Decision Tree achieved the highest performance with a cross-validated ROC-AUC of around 0.9475. This marks the first time that every possible combination of three classifiers has been exhaustively evaluated among the 220 possibilities, and the same three-classifier combination was found with the same ROC-AUC as a deterministic benchmark. The classifiers were then used to build the final equal-weight soft-voting ensemble.

### 4.6 Soft voting ensemble performance

The classifiers selected by the genetic algorithm (Decision Tree, XGBoost, LightGBM) were fused using soft voting. The ensemble achieved an accuracy of 0.8799, precision of 0.8717, recall of 0.8907, specificity of 0.8692, F1-score of 0.8811, and ROC-AUC of 0.9490. Compared to the best individual classifier, LightGBM, the ensemble demonstrated modestly higher point estimates for accuracy, precision, recall, specificity, and F1-score, whereas LightGBM achieved a marginally higher ROC-AUC (0.9498 vs. 0.9490). The highest point estimates for all measures (accuracy, recall, specificity, and F1-score) were obtained with the soft voting ensemble among the candidate models selected by the genetic algorithm. Figure 5 provides a performance comparison of the ensemble model. Table 5 presents a performance comparison of the top three selected models and the final ensemble model.

**Figure 5:**
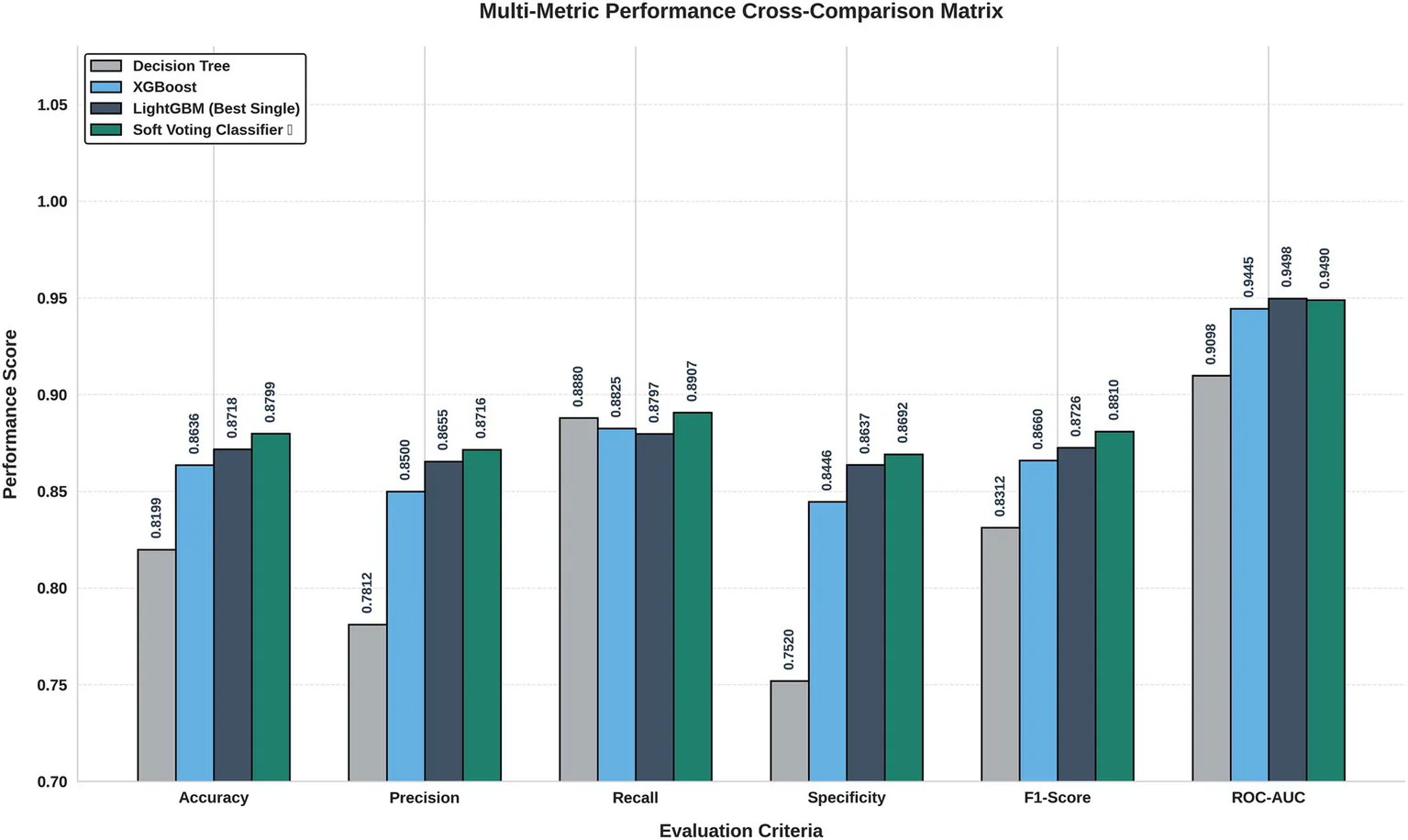
Multi-metric performance cross-comparison chart. Decision Tree, XGBoost, LightGBM, and the soft voting ensemble were compared across accuracy, precision, recall, specificity, F1-score, and ROC-AUC.

**Table 5:** Performance comparison of the GA-selected candidate models and the final ensemble.

Model	Accuracy	Precision	Recall	Specificity	F1-score	ROC-AUC
Decision Tree (DT)	0.8199	0.7813	0.8880	0.7520	0.8312	0.9098
XGBoost (XGB)	0.8636	0.8500	0.8825	0.8447	0.8660	0.9445
LightGBM (LGBM)	0.8718	0.8656	0.8798	0.8638	0.8726	0.9498
Soft Voting	0.8799	0.8717	0.8907	0.8692	0.8811	0.9490

The ROC analysis showed good discriminative power in all GA-selected models and the calibrated ensemble. Figure 6 illustrates the ROC-AUC values for each individual classifier and the ensemble, showing that LightGBM had the largest AUC value, while the ensemble remained at a similar value. The ensemble curve consistently stays above the reference line and outperforms XGBoost and Decision Tree in terms of the other metrics shown in Table 5, reflecting its strong ability to preserve global ranking performance while enhancing other metrics.

**Figure 6:**
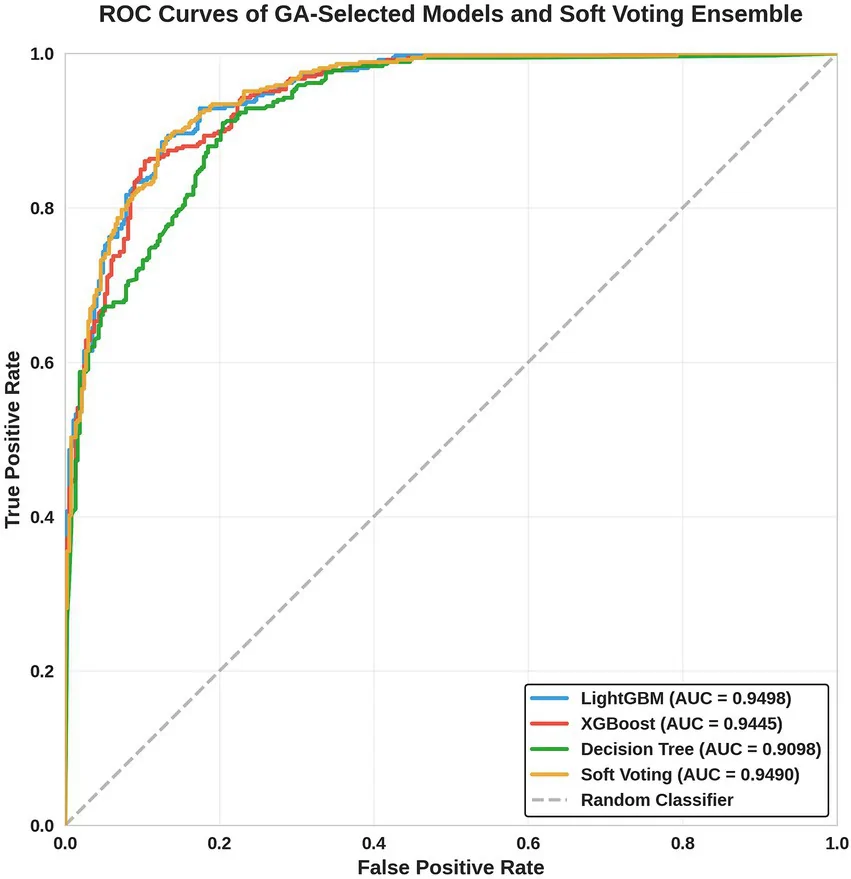
ROC curves of GA-selected models and the soft voting ensemble. Receiver operating characteristic curves are shown for LightGBM, XGBoost, Decision Tree, and the soft voting ensemble on the held-out test set.

Under the implemented encoding (sensitive = the positive class), the confusion matrix yielded 319 true negatives, 48 false positives, 40 false negatives, and 326 true positives. Therefore, the true positive cases are those that are correctly predicted as sensitive, and the true negative cases are those that are correctly predicted as resistant. The sensitivity and specificity values are balanced, indicating stable performance on both classes. The confusion matrix distribution of the soft voting ensemble is shown in Figure 7.

**Figure 7:**
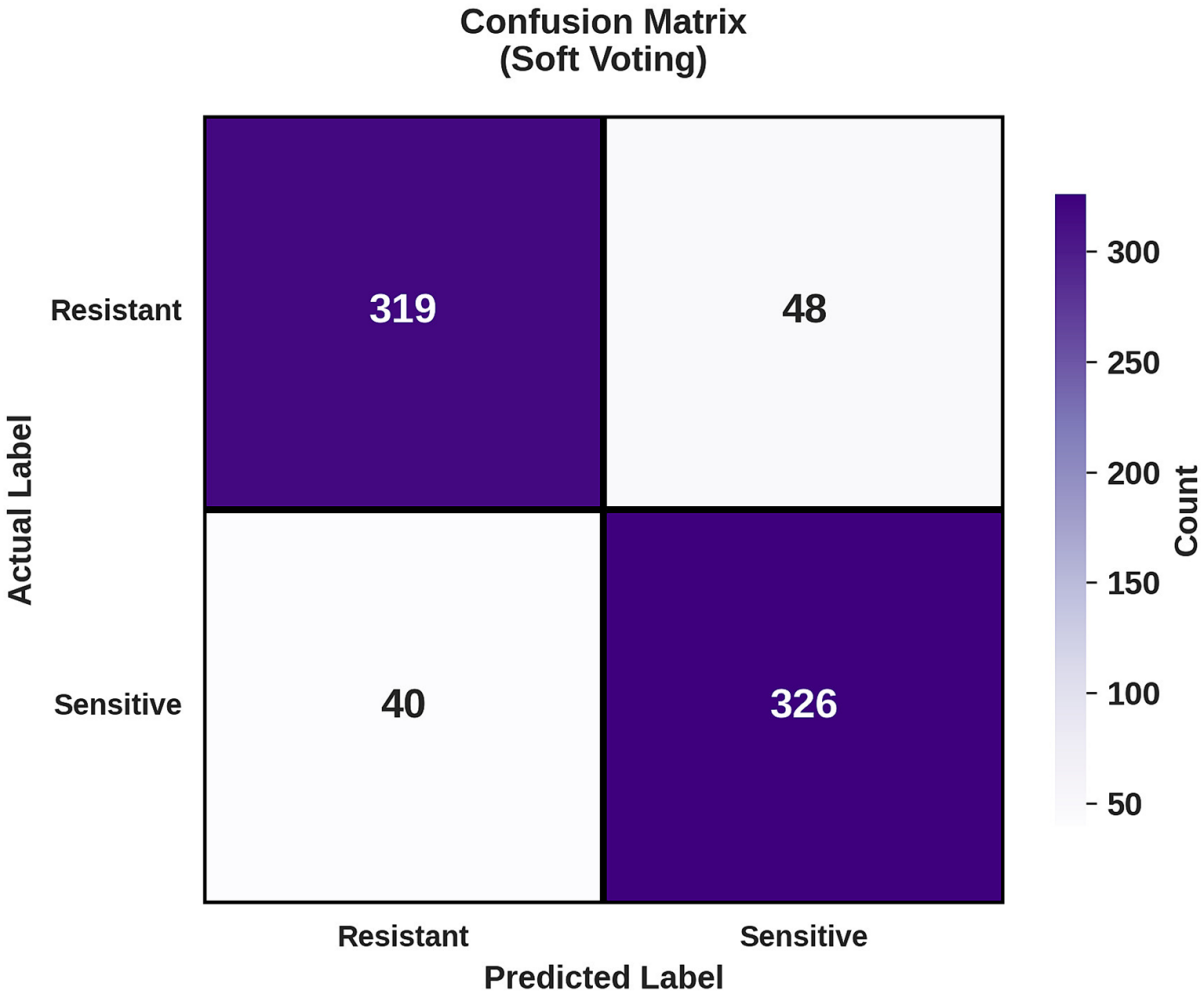
Confusion matrix of the soft voting ensemble.

### 4.7 Statistical validation

The soft-voting ensemble after calibration was statistically validated to be stable and reliable. The five-fold cross-validation procedure yielded consistent performance with an ROC-AUC of 0.9405 ± 0.0101 and an accuracy of 0.8621 ± 0.0195, demonstrating good generalization. The model’s performance on the test set achieved an accuracy of 0.8799, recall of 0.8907, specificity of 0.8692, and an F1-score of 0.8811. Sensitive = 1 (recall) and Resistant case = 1 (specificity). The performance of the ensemble and LightGBM are not significantly different using DeLong’s test (ΔAUC = 0.0007, *p* = 0.5906), and McNemar’s test (*p* = 1.0000) shows that the classification errors are not significantly different between them. Stable and reliable performance was further confirmed by the use of confidence intervals as well as Cohen’s kappa (κ = 0.7599). Table 6 summarizes the statistical validation of the proposed ensemble model using five complementary validation pillars.

**Table 6:** Comprehensive statistical validation results of the proposed voting ensemble classifier.

Validation pillar	Statistical test	Key results	Interpretation
Generalization	5-fold stratified cross-validation	ROC-AUC: 0.9405 ± 0.0101; Accuracy: 0.8621 ± 0.0195; Sensitivity: 0.8632 ± 0.0224; Specificity: 0.8611 ± 0.0289; F1-score: 0.8620 ± 0.0190	Stable performance (An average score across folds)
Held-out performance	Test-set evaluation	Accuracy: 0.8799; Sensitivity: 0.8907; Specificity: 0.8692; F1-score: 0.8811	Balanced classification performance
Global discrimination	DeLong test	Ensemble AUC: 0.9490; LightGBM AUC: 0.9483; ΔAUC: 0.0007; *p* = 0.5906	Similar discriminative performance as baseline
Classification consistency	McNemar’s test	Agreement: 728/733; discordant pairs: 3 vs. 2; *p* = 1.0000	There is no significant difference in the number of classification errors.
Uncertainty estimation	Wilson & bootstrap confidence intervals	Sensitivity CI: 0.8546–0.9187; Specificity CI: 0.8309–0.8999; ROC-AUC CI: 0.9350–0.9625	Stable uncertainty bounds

### 4.8 SHAP-based explainability analysis

Interpretation of the contribution of selected predictors to the model prediction was carried out using SHAP-based explainability analysis. The SHAP beeswarm plot shown in Figure 8 for LightGBM, the best individual model, was used to explore the effects of features in the global test set.

**Figure 8:**
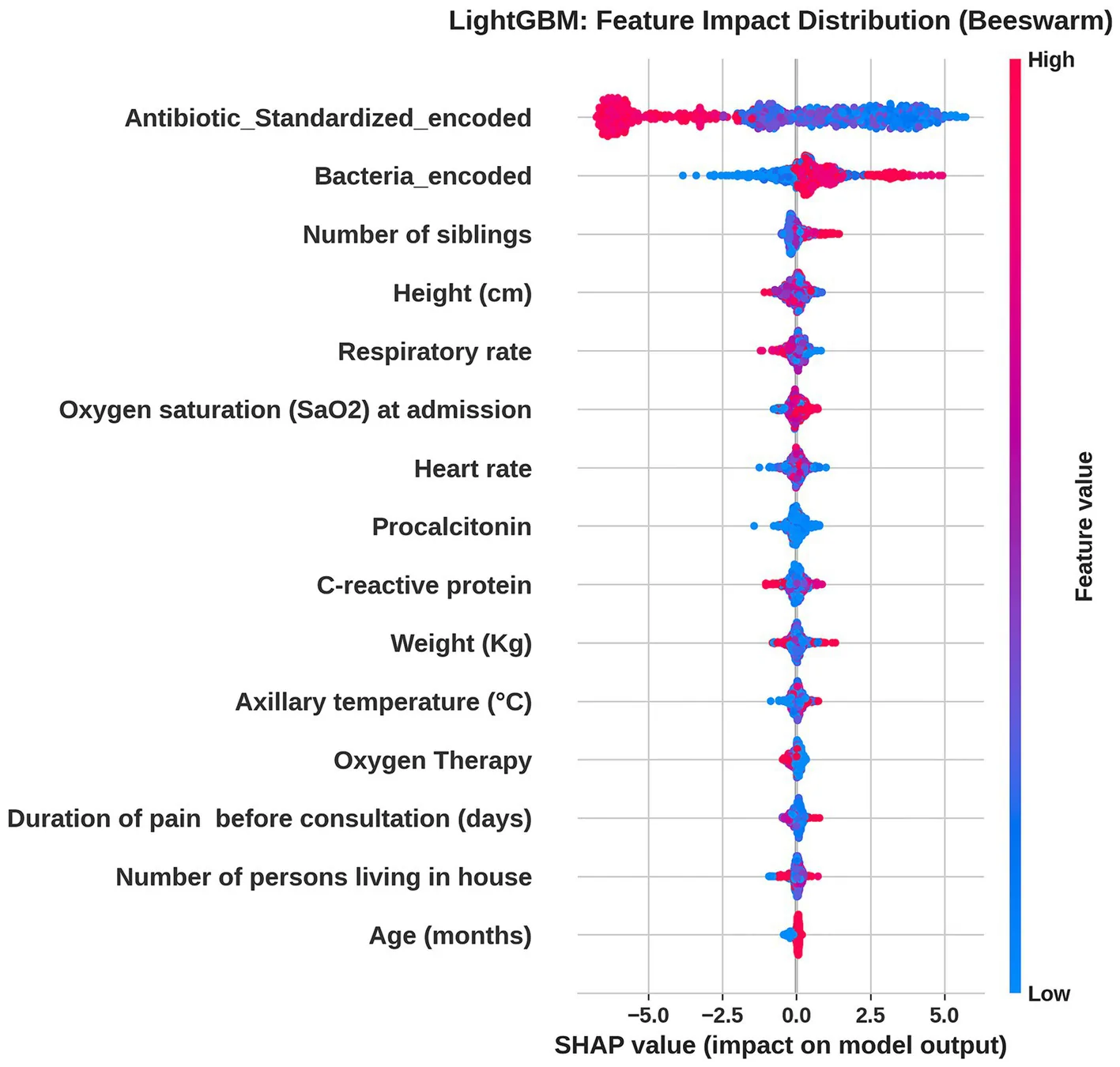
SHAP beeswarm plot of the LightGBM model that illustrates feature contributions for samples in the test set. The color of each point corresponds to the value of the features, and the position corresponds to the SHAP importance with respect to the model output.

The largest SHAP attribution magnitudes were for the encoded antibiotic identity and the encoded bacterial organism (Figure 8). This is not surprising since the susceptibility evaluation is done with respect to the tested antimicrobial agent and the bacterial organism in question. Other contextual predictors, such as clinical, physiological, and inflammatory factors, also contributed to the SHAP results, but the SHAP values are not direct measures of the additional predictive value of each predictor. If the implemented outcome coding (sensitive = 1) is used, a positive SHAP value indicates a contribution to the sensitive class, and a negative value indicates a contribution to the resistant class. The antibiotic and bacterial variables were encoded as categorical model attribution (not clinical) effects. For the ensemble model, the ensemble-weighted SHAP ranking was performed by summing the individual model SHAP rankings with the weights from the soft voting.

The ensemble-level ranking shown in Figure 9 verified that antibiotic and bacterial features were the most important in the final model interpretation and indicated that selected clinical and environmental features were the next most important. These results align with the feature selection findings, lending credence to the framework’s interpretability. No causal interpretations were made of the SHAP values; rather, they were treated as *post hoc* attributions.

**Figure 9:**
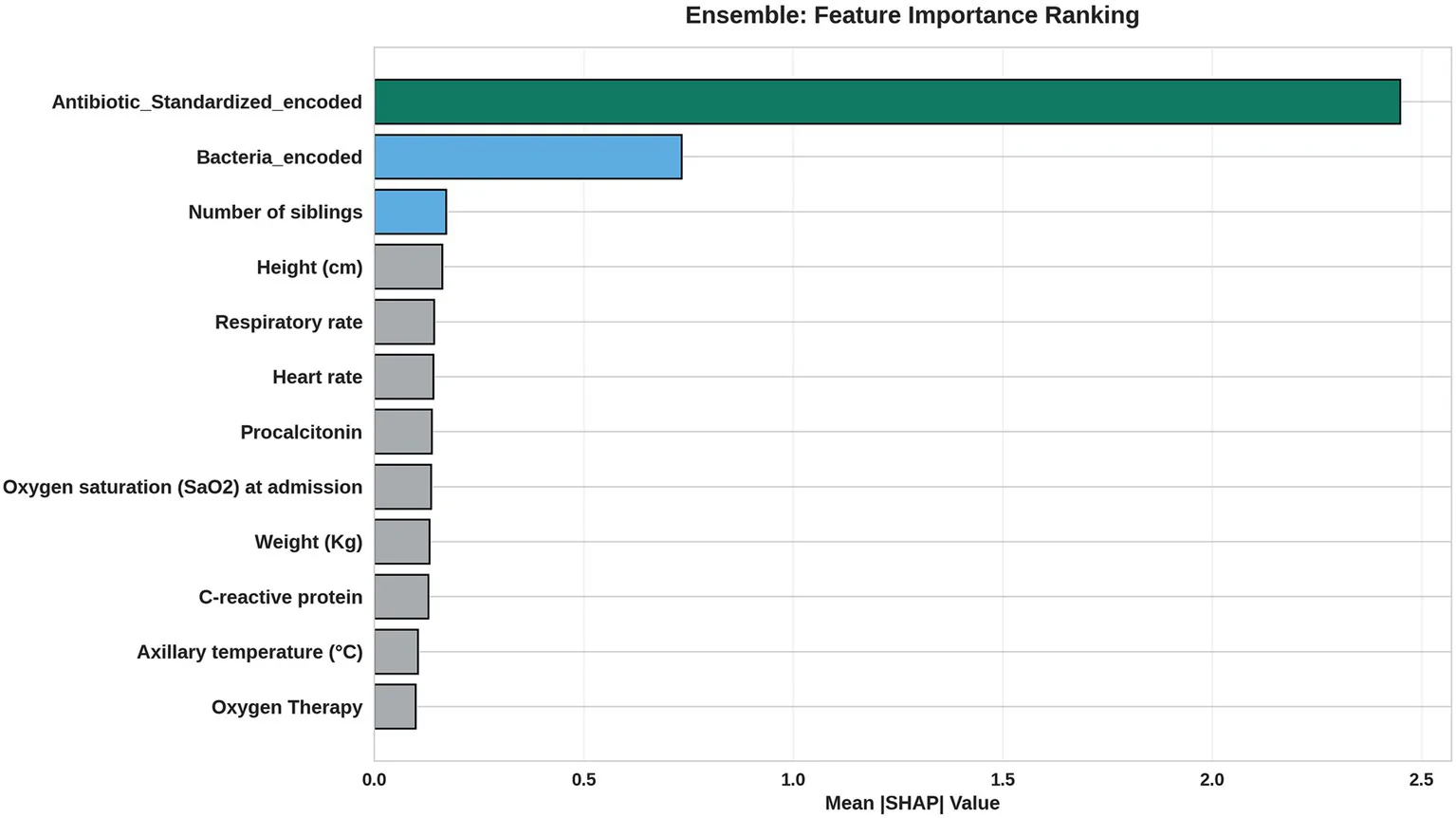
The soft voting model feature importance ranking by the ensemble SHAP method, showing that antibiotic and bacterial features are the most important, followed by clinical and environmental features.

### 4.9 Clinical and methodological interpretation

The results show that organism–antibiotic interactions play a major role in predicting antibiotic susceptibility in children, with a secondary contribution from clinical, inflammatory, anthropometric, environmental, and hospitalization-related variables. The microbiological basis of antimicrobial susceptibility is reflected in the dominant importance of encoded antibiotic and bacterial features in the feature selection and SHAP analyses. In addition to microbiological variables, patient-related variables also made a significant contribution to model prediction. Physiological parameters like heart rate, respiratory rate, oxygen saturation, and temperature are markers of recent clinical conditions, while inflammatory parameters like C-reactive protein and procalcitonin represent the burden of infection. Age, height, and weight further illustrate the patient-level heterogeneity of pediatric populations as anthropometric variables.

Household variables may be related to exposures, and radiological and hospitalization-related variables (e.g., chest X-ray results, oxygen therapy, length of stay) help provide clinical context. However, these features should be interpreted with care, as some can relate to the stage of the disease and/or treatment intensity and are not necessarily indicative of early-stage prediction. From a methodological point of view, the results allow for implementing a multi-agent feature selection framework. Embedded, filter, and wrapper schemes were able to find complementary relevance patterns, while consensus weighting and redundancy penalization facilitated the selection of a small and consistent subset of features. The calibrated soft voting ensemble showed the best threshold-dependent measures, with discriminative performance close to that of the highest individual model. In summary, the developed framework offers a coherent and statistically sound means for predicting pediatric antibiotic susceptibility that is understandable.

### 4.10 Ablation study

An ablation study was conducted by comparing the proposed Top-20 feature representation with the complete 64-feature representation to evaluate the contribution of the proposed feature selection strategy. The genetic algorithm (GA) was separately executed for each feature representation with the same GA configuration and optimization parameters (same chromosome structure, three-classifier selection constraint, fitness criterion, and evolutionary parameters). Therefore, both feature representations were tested against an equivalent model selection protocol, except for the feature representation on the input side of the ablation settings. Table 7 shows the performance of individual classifiers and GA-selected soft voting ensembles for the 64 and Top-20 features.

**Table 7:** Performance of the individual classifiers and corresponding GA-selected soft-voting ensembles using the 64-feature and Top-20 feature representations.

Model	Features	Accuracy	Precision	Recall	F1-score	ROC-AUC
Decision Tree (DT)	64	0.7789	0.7040	0.9617	0.8129	0.8640
Extreme Gradient Boosting (XGB)	64	0.7885	0.8661	0.7683	0.8130	0.8171
Random Forest (RF)	64	0.7762	0.7095	0.9344	0.8066	0.8180
Soft Voting (DT + XGB + RF)	64	0.8281	0.7955	0.8825	0.8367	0.9215
LightGBM (LGBM)	20	0.8718	0.8656	0.8798	0.8726	0.9498
Extreme Gradient Boosting (XGB)	20	0.8636	0.8500	0.8825	0.8660	0.9445
Decision Tree (DT)	20	0.8199	0.7813	0.8880	0.8312	0.9098
Soft Voting (DT + XGB + LightGBM)	20	0.8799	0.8717	0.8907	0.8811	0.9490

The genetic algorithm was independently applied to each feature representation to identify the optimal three-classifier ensemble.

Table 7 shows that the Top-20 representation had superior predictive performance compared to the 64-feature representation. The GA selected a soft-voting ensemble of Decision Tree (DT), XGBoost (XGB), and Random Forest (RF) classifiers for the 64-feature representation, which achieved an accuracy of 0.8281, a precision of 0.7955, a recall value of 0.8825, an F1-score of 0.8367, and an ROC-AUC value of 0.9215. By comparison, the GA was able to select DT, XGB, and LightGBM (LGBM), which achieved an accuracy of 0.8799, precision of 0.8717, recall of 0.8907, an F1-score of 0.8811, and an ROC-AUC of 0.9490.

Table 8 presents a comparison of the performance of the GA-selected ensembles using 64-feature and Top-20 representations. Compared to the 64-feature representation, the accuracy of the Top-20 representation improved by 5.18 percentage points, with precision increasing by 7.62 percentage points, recall by 0.82 percentage points, F1-score by 4.44 percentage points, and ROC-AUC by 2.75 percentage points. The improvement in ROC-AUC indicates an overall enhancement in discriminative ability, while the increases in accuracy, precision, and F1-score reflect improved classification performance. The ROC-AUC comparison of the Top-20 and Top-64 feature representations is shown in Figure 10.

**Table 8:** Comparison of the performance of the GA-selected ensembles using the 64-feature and Top-20 feature representations.

Feature representation	GA-selected ensemble	Accuracy	Precision	Recall	F1-score	ROC-AUC
64-Feature	(DT + XGB + RF)	0.8281	0.7955	0.8825	0.8367	0.9215
Top-20	(DT + XGB + LGBM)	0.8799	0.8717	0.8907	0.8811	0.9490

The genetic algorithm was independently applied to each feature representation to identify the optimal three-classifier ensemble.

**Figure 10:**
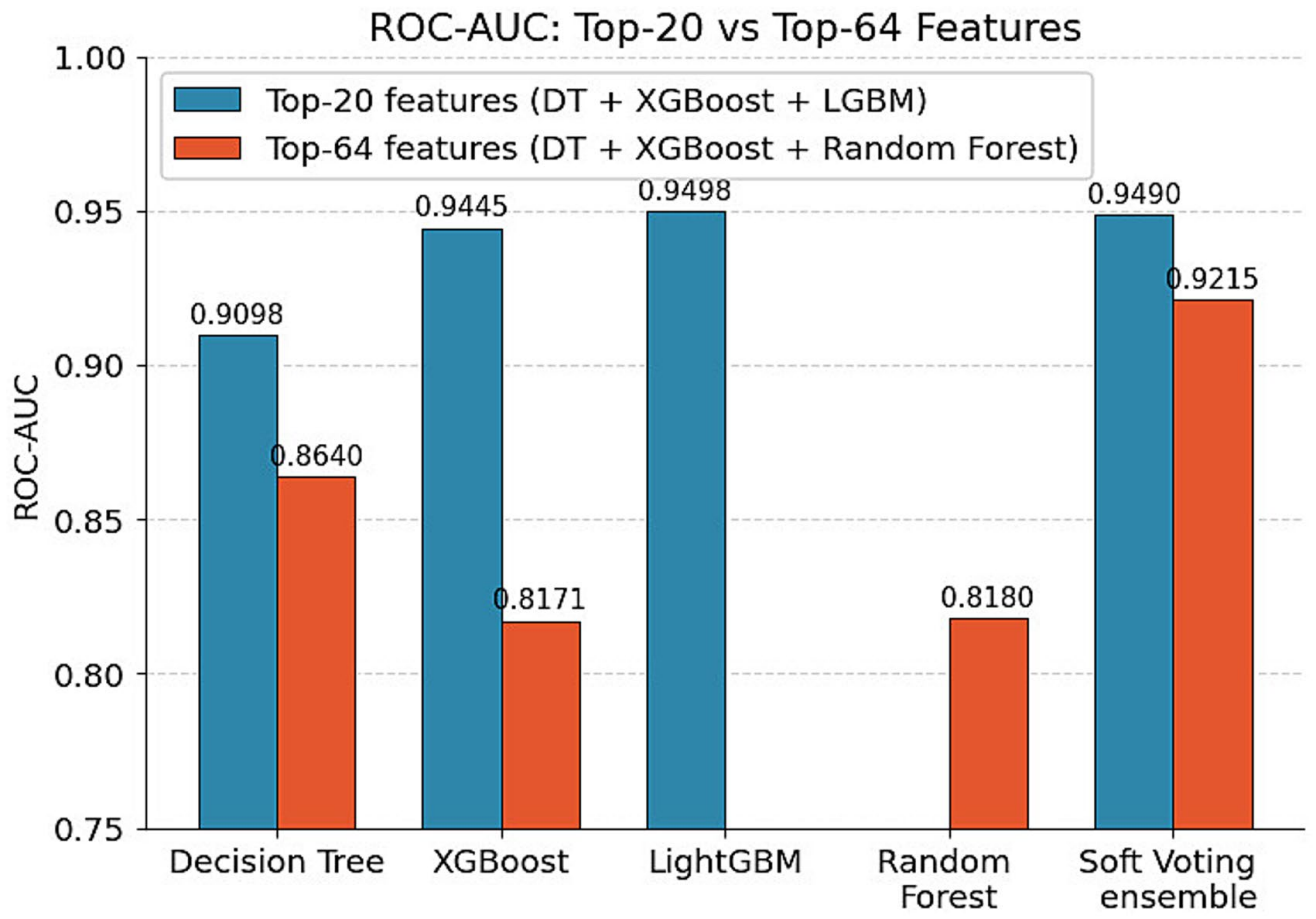
ROC-AUC comparison of Top-20 and Top-64 feature representations.

### 4.11 Strengths and limitations

Overall, the study presents several strengths, including the integration of embedded, filter-based, and wrapper methods within the consensus-weighted multi-agent feature selection framework, the use of a comprehensive modeling pipeline involving multiple machine learning algorithms, genetic algorithm-based model selection, ensemble learning, statistical validation, and SHAP-based interpretability.

Nevertheless, some important limitations should be acknowledged. The train–test partition was performed at the patient–bacterium–antibiotic record level rather than using patient-grouped splitting. Future studies should use patient-grouped partitioning and external multi-center validation to further assess generalizability. The cross-validation results reflect performance conditional on the selected Top-20 feature representation. The present study did not directly assess the incremental predictive value of clinical, inflammatory, anthropometric, environmental, and healthcare-related variables beyond antibiotic identity and bacterial organism. Although these variables were selected by the feature selection framework and contributed to predictions in the SHAP analysis, this does not by itself demonstrate improved predictive performance over an organism–antibiotic-only baseline model. An additional limitation is the absence of independent external validation. Therefore, the findings require confirmation through external, multi-center, and prospective validation before any consideration of clinical deployment.

### 4.12 Sustainable development goal alignment

The proposed framework is conceptually consistent with Sustainable Development Goal 3 (Good Health and Well-Being) as it aims to predict the risk of antibiotic susceptibility in pediatric respiratory infections using data, along with its potential applicability to research on antimicrobial stewardship. By integrating transparent feature selection, redundancy control, statistical validation, ensemble learning, and SHAP-based interpretability, the framework provides a methodological basis for future development of interpretable antimicrobial decision-support approaches. However, the present study represents an internally validated machine learning investigation and does not establish clinical effectiveness, external generalizability, or readiness for deployment. Thus, any possible contributions to antimicrobial decision support, rational use of antibiotics, or clinical management must be viewed as prospective and require validation through independent external, multi-center, and prospective studies.

### 4.13 Future directions

First, future studies should perform controlled feature-set ablation comparing models based solely on bacterial organisms and antibiotic identities with models using the complete selected feature set. Using identical downstream classifiers and evaluation protocols would allow the incremental predictive value of clinical, inflammatory, anthropometric, environmental, and healthcare-related variables, beyond the dominant organism–antibiotic predictors, to be quantified.

The following possible extensions could further improve the proposed framework. First, future work can examine the automated synthesis of features with medical large language models (Med-LLMs) that extract information from unstructured clinical narratives. This could be used in addition to the existing multi-agent feature selection, which can incorporate latent textual information into the predictive pipeline. Second, an existing interpretability framework based on SHAP can be expanded to include counterfactual explanation methods. These methods might provide actionable information by identifying the minimal modifications that could alter forecasts to lower-risk scenarios. Third, the framework can be enhanced with approaches that protect sensitive patient data, including federated learning, which enables multi-institutional model development without sharing patients’ data. Furthermore, particularly for real-world clinical integration, future studies should consider uncertainty-aware deployment strategies, such as monitoring calibration drift and local epidemiological changes.

## 5 Conclusion

In this study, a consensus weighted multi-agent feature selection framework with redundancy penalization was proposed to predict the antibiotic susceptibility of pediatric samples using clinical and antibiogram information. The framework identified a small and non-redundant panel of 20 predictors across microbiological, clinical, and environmental domains by combining six complementary feature selection approaches. The ensemble method of soft voting outperformed some metrics such as accuracy, recall, specificity, F1-score, and Matthews correlation coefficient compared to LightGBM, which excelled on individual metrics. The statistical validation of the ensemble demonstrated that it remained discriminative and exhibited stable and reliable model behavior similar to LightGBM. Analysis of the importance of individual predictions revealed that organism–antibiotic interactions were the primary driver, while other clinical and inflammatory features also significantly contributed to predicting the organism–antibiotic interaction. Overall, the framework offers a statistically well-supported and interpretable method for predicting antibiotic susceptibility.

## Data Availability

Publicly available datasets were analyzed in this study. This data can be found here: https://data.mendeley.com/datasets/2d9dvnycjw/1.
